# ctDNA and immune biomarker testing for molecular residual disease surveillance and immune stratification in cancer

**DOI:** 10.3389/fmed.2026.1942673

**Published:** 2026-09-09

**Authors:** Ning Cui, Hongying Hao, Xiaofeng Hu, Yaxuan Li

**Affiliations:** Huanghe Science and Technology University, Zhengzhou, Henan, China

**Keywords:** cancer immunotherapy, circulating tumor DNA, immune biomarker, immune stratification, laboratory diagnostics, liquid biopsy, molecular residual disease, recurrence surveillance

## Abstract

Molecular residual disease (MRD) refers to occult cancer persisting below the detection limit of conventional imaging and is a major source of later relapse. Circulating tumor DNA (ctDNA) provides a minimally invasive molecular surrogate of residual disease, but its interpretation is influenced by tumor burden, shedding, anatomic site, treatment-related cell death, sampling time, and analytical sensitivity. Immune biomarkers provide complementary information on whether residual disease is being recognized, contained, or permitted to escape by the host immune system. This Mini Review examines how ctDNA and immune biomarker testing can be integrated for MRD surveillance and immune stratification across solid tumors. We summarize tumor-informed and tumor-naïve ctDNA approaches, including mutation-, methylation-, fragmentomic-, and whole-genome-based assays; discuss tissue and blood immune biomarkers such as PD-L1, mismatch repair status, tumor mutational burden, antigen-presentation integrity, T-cell receptor repertoires, cytokines, and suppressive myeloid populations; and review recent evidence from colorectal, lung, urothelial, breast, and melanoma studies. We propose a conceptual, hypothesis-generating framework in which ctDNA reports tumor-derived molecular signal and shedding, whereas immune assays report immune pressure and escape. Joint interpretation may, if prospectively validated, distinguish biologically different MRD states, improve longitudinal risk assessment, and provide a laboratory foundation for biomarker-oriented immunotherapy and cell-therapy studies. However, clinical implementation requires standardized preanalytics, explicit assay limits, paired leukocyte controls, serial sampling, harmonized immune assays, and prospective trials demonstrating clinical utility rather than prognostic association alone.

## 1 Introduction

After apparently curative surgery, radiotherapy, or multimodal treatment, a proportion of patients retain microscopic disease that is not visible on standard imaging. The term molecular residual disease emphasizes that this state is inferred from tumor-derived molecular signals rather than from measurable lesions. ctDNA is attractive because it can be sampled repeatedly and can report tumor-specific genomic or epigenomic material in plasma. Yet ctDNA alone cannot determine whether residual cells are immunologically visible, whether an antitumor response is active, or whether immune escape has already occurred. Whether immune profiling adds reproducible clinical value beyond ctDNA alone remains a central unresolved question.

The same limitation applies in the opposite direction. Established and emerging immunotherapy biomarkers—including PD-L1, mismatch repair deficiency or microsatellite instability, tumor mutational burden, immune gene-expression programs, antigen-presentation machinery, and circulating immune-cell states—describe the tumor–host interface but do not prove that viable residual cancer is present (1–6). Response and resistance to immune checkpoint blockade are determined by interacting tumor-intrinsic and host factors rather than by a single marker (7, 8). Even accessible systemic indices such as the neutrophil-to-lymphocyte ratio acquire greater meaning when interpreted with tumor genomic features rather than alone (9). This Mini Review therefore treats ctDNA and immune biomarker testing as orthogonal laboratory measurements. The central proposition is that ctDNA provides a molecular surrogate of residual disease and shedding, whereas immune biomarkers estimate immune recognition, effector competence, and escape.

## 2 ctDNA as a molecular measure of residual disease

ctDNA constitutes a variable fraction of total cell-free DNA and carries tumor-derived sequence, methylation, copy-number, and fragmentation features. Current MRD assays are broadly tumor-informed or tumor-naïve. Tumor-informed assays first sequence the resected tumor and then track a personalized set of variants in plasma. Their major advantage is high specificity, especially when many patient-specific variants are monitored. Tumor-naïve assays avoid the need for tissue and may combine recurrent genomic alterations with methylation or fragmentomic signatures, improving turnaround time and applicability when tissue is unavailable. European recommendations emphasize that ctDNA assays should be used with explicit attention to analytical validity, clinical context, and the distinction between prognostic evidence and proven clinical utility (10). The two strategies differ in tissue requirements, assay personalization, genomic breadth, turnaround time, specificity, and vulnerability to background or clonal-hematopoiesis signals (Table 1).

**Table 1:** Concise comparison of tumor-informed and tumor-naïve ctDNA approaches for molecular residual disease testing.

Feature	Tumor-informed approach	Tumor-naïve approach
Tumor tissue	Usually required for assay design	Not required
Assay architecture	Personalized tracking of patient-specific tumor variants	Fixed genomic, methylation, fragmentomic, or genome-wide features
Principal strength	High specificity when multiple confirmed tumor variants are tracked	Faster deployment and applicability when tissue is unavailable
Principal limitation	Tissue dependence, personalization time, assay failure, and clonal evolution	Background signals, clonal hematopoiesis, model dependence, and variable specificity
Sensitivity determinants	Number of variants, plasma input, depth, residual burden, and shedding	Feature breadth, training/validation design, plasma input, residual burden, and shedding
Reporting priorities	Tracked variants, plasma volume, limit of detection, leukocyte control, and positivity rule	Feature set, algorithm, threshold, calibration, plasma volume, and leukocyte filtering

MRD concentrations can approach parts-per-million levels, so error suppression is decisive. Benchmarking studies have shown substantial variability among sequencing assays, particularly at low variant allele fractions (11). Experimental workflow factors—including plasma volume, DNA input, unique molecular identifiers, depth, background error modeling, and sample-level positivity rules—can materially alter sensitivity and specificity (12). Recent platforms seek to expand the number of informative molecules without sacrificing specificity: MAESTRO-Pool enriches large panels of patient-specific variants, whereas whole-genome approaches with single-read error correction extend detection beyond targeted panels (13, 14). These innovations address sensitivity, but they do not remove biological constraints such as low tumor shedding, sanctuary sites, small residual deposits, or intermittent release.

Serial testing is generally more informative than a single landmark sample because persistent positivity, new conversion to positivity, and durable clearance represent different biological trajectories. However, trials differ in sampling windows, assay thresholds, and definitions of clearance. Reviews of ctDNA-guided adjuvant trials have therefore stressed the importance of trial design, including prespecified intervention rules and appropriate control groups (15). Negative ctDNA should be reported as “not detected within the analytical and biological limits of the assay,” not as proof of absence of disease. A transient early increase can reflect treatment-induced cell death, whereas persistent or rising ctDNA more strongly supports ongoing disease; durable clearance and later re-emergence should be interpreted as distinct longitudinal states. Recent ASCO guidance likewise emphasizes that ctDNA should complement rather than replace standard clinical assessment and that MRD-guided treatment decisions require evidence-based settings or clinical trials (16).

## 3 Immune biomarkers that contextualize ctDNA-defined MRD

Tumor-intrinsic immune biomarkers indicate whether residual cancer can be recognized and attacked. PD-L1 expression reflects an adaptive or constitutive checkpoint state, whereas MSI-high or dMMR status has an established indication-specific role in selected settings. High TMB may indicate greater mutational and neoantigen potential, but it is not a direct measure of neoantigen quality, antigen presentation, or immune activation and is sensitive to assay platform, panel size, filtering, and threshold. These variables remain incomplete because antigen quantity does not guarantee presentation. Defects in HLA class I, B2M, TAP1/TAP2, or related antigen-presentation machinery are associated with reduced benefit from checkpoint blockade and can create an immune-invisible residual clone (17). Thus, ctDNA positivity in a tumor with preserved antigen presentation is biologically different from ctDNA positivity accompanied by HLA/B2M disruption.

Tissue and blood measurements also capture different immune compartments. Multiplex tissue analyses can resolve intratumoral CD8-positive, LAG-3-positive, PD-1-positive, and myeloid populations, whereas peripheral assays provide serial access to systemic immunity. A urothelial carcinoma study integrating blood and tissue biomarkers showed that the two compartments supplied complementary rather than interchangeable information (18). In melanoma, circulating immune-cell bioenergetics and metabolomic features were associated with response to anti-PD-1 therapy, supporting the concept that functional peripheral assays can add information beyond cell counts (19). Peripheral measurements cannot be assumed to represent the immune microenvironment at the residual-disease site. PD-L1, TILs, HLA/B2M expression, and immune-cell composition may also differ between the primary tumor, metastatic or residual sites, and pre- vs. post-treatment samples.

Accessible immune biomarkers include cytokine profiles, suppressive myeloid populations, and composite inflammatory indices. A recent systematic review found associations between circulating cytokines and checkpoint-blockade outcomes, but assay heterogeneity and nonuniform cutoffs limited translation (20). A meta-analysis of circulating myeloid-derived suppressor cells showed that lower MDSC levels, particularly monocytic MDSCs, were associated with better outcomes during immune checkpoint therapy (21). Similarly, pan-immune inflammation value and related blood-cell indices are reproducible prognostic markers, although they are not specific measures of antitumor immunity (22).

T-cell receptor sequencing offers a more direct view of adaptive immune dynamics. Peripheral repertoire expansion and sharing with tumor-infiltrating clones have been associated with durable anti-PD-1 responses in renal cell carcinoma (23), while baseline diversity and repertoire similarity showed predictive associations in gastrointestinal cancers (24). Sequence-aware machine-learning approaches can extract repertoire features that are not captured by simple clonality metrics (25). For integrated MRD research, TCR sequencing should be regarded as an emerging complementary biomarker of immune response rather than an established assay for MRD detection or surveillance.

## 4 Mechanistic integration of residual burden and immune state

Microscopic residual cancer exists within a dynamic balance between tumor-cell survival and immune pressure. Residual cells release ctDNA through apoptosis, necrosis, and possibly active secretion. The amount detected in plasma therefore reflects both the number of viable cells and their rate of turnover. An immune-active residual niche may generate tumor killing and transient DNA release, whereas a progressively rising ctDNA signal more strongly suggests expanding burden. Conversely, an immune-suppressed niche can permit persistent disease despite weak inflammatory or lymphocyte signals. The plasma ctDNA signal is therefore a molecular surrogate influenced by viable burden and shedding biology rather than a direct count of residual tumor cells.

**Figure 1:**
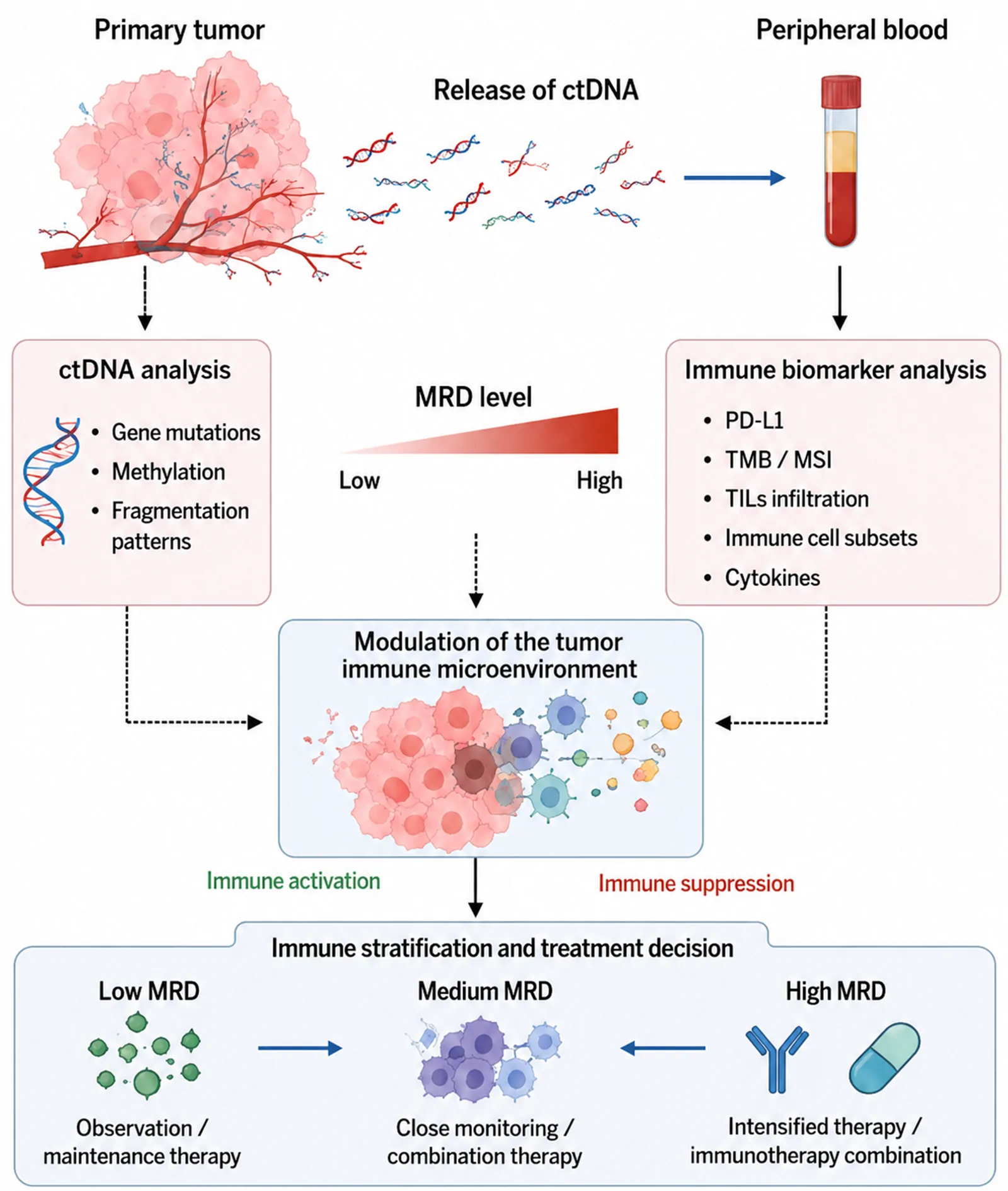
Mechanistic coupling of ctDNA release and immune-state biomarkers in molecular residual disease. Microscopic residual tumor cells release ctDNA through cell death and turnover, generating genomic, methylation, fragmentation, and kinetic signals in peripheral blood. In parallel, antigen presentation and CD8-positive T-cell activity impose immune pressure, whereas PD-1/PD-L1 signaling, HLA/B2M loss, TGF-*β*/IL-10 signaling, MDSCs, and regulatory T cells enable persistence. ctDNA therefore reports residual burden and shedding, while immune biomarkers report immune recognition, competence, and escape.

Immune escape can occur at several mechanistic levels. PD-1/PD-L1 engagement restrains effector-cell function; TGF-*β*, IL-10, regulatory T cells, and MDSCs suppress or exclude cytotoxic lymphocytes; and HLA/B2M loss prevents effective antigen display. These mechanisms explain why the same ctDNA signal may carry different biological implications. On this basis, we propose four hypothesis-generating states: ctDNA detected with immune recognition; ctDNA detected with immune suppression or escape; ctDNA not detected with immune activity; and ctDNA not detected with immune quiescence. These states have not been prospectively validated as a clinical classification and should be interpreted with assay-specific limits, tumor shedding, treatment timing, and longitudinal confirmation (Table 2 and Figure 1).

**Table 2:** Proposed ctDNA-immune states and their current level of evidence.

Proposed state	Biological interpretation	Current evidence status	Key caveat
ctDNA detected/immune-active	Tumor-derived signal with evidence of immune recognition	Mechanistic and limited translational support; no prospective validation	Kinetics may reflect burden or treatment-induced killing
ctDNA detected/immune-suppressed	Tumor-derived signal with escape, exclusion, or impaired presentation	Mechanistic support; clinical classification unvalidated	Requires tissue or functional immune context
ctDNA not detected/immune-active	Possible clearance, low burden, or low shedding with immune activity	Hypothesis-generating	A negative result does not exclude disease
ctDNA not detected/immune-quiescent	Limited evidence of tumor-derived signal or active immunity	Most hypothetical state	Requires repeat testing and disease-specific interpretation

## 5 Evidence across tumor types and immunotherapy settings

Colorectal cancer provides the strongest contemporary evidence for postoperative ctDNA MRD. In the randomized DYNAMIC study, a ctDNA-guided strategy reduced adjuvant chemotherapy use in stage II colon cancer without compromising recurrence-free survival (26). The GALAXY observational program showed strong prognostic stratification and treatment-associated outcome differences, but it was not a randomized biomarker-guided trial (27). Updated analyses in more than 2,000 patients reinforced the association between ctDNA-defined MRD, disease-free survival, and overall survival, and distinguished sustained from transient clearance (28). Plasma-only assays integrating genomic and epigenomic signals have shown high specificity, while serial testing improves sensitivity (29). The UK TRACC Part B study and a tumor-naïve GALAXY analysis further support tissue-free genomic–methylation strategies for postoperative surveillance (30, 31).

In resectable non-small cell lung cancer, TRACERx demonstrated that ctDNA can track residual disease, metastatic dissemination, and subclonal architecture (32). Perioperative studies found that postoperative ctDNA positivity was a strong recurrence marker and could precede radiographic relapse (33–35). In patients receiving neoadjuvant immunotherapy or chemoimmunotherapy, ctDNA dynamics were concordant with pathological response and postoperative recurrence risk, although available cohorts remain relatively small (36). These studies show why an immune-context layer is relevant: ctDNA clearance may reflect both cytoreduction and immune-mediated elimination, whereas persistence can result from inadequate immune recognition, residual resistant clones, or insufficient assay coverage.

The clearest direct connection between ctDNA-defined MRD and immunotherapy comes from urothelial carcinoma. An exploratory ctDNA analysis of IMvigor010, whose parent phase 3 trial did not meet its primary efficacy endpoint, suggested differential atezolizumab benefit among postoperative ctDNA-positive patients; this finding requires prospective validation before it can support routine ctDNA-guided immunotherapy selection (37). Across advanced solid tumors treated with immune checkpoint inhibitors, a systematic review found that early ctDNA reduction or clearance was associated with substantially better progression-free and overall survival (38). A prospective NSCLC study combining NK-cell activity with methylated HOXA9 ctDNA showed that joint tumor and immune measurements produced stronger prognostic separation than either dimension alone (39).

Other tumor types support the generalizability of longitudinal MRD detection while also illustrating assay dependence. Bespoke ctDNA identified high-risk melanoma patients before clinical relapse (40). In early triple-negative breast cancer, personalized multimutation sequencing detected MRD earlier than digital PCR in the cTRAK-TN cohort (41). Serial tumor-informed monitoring in high-risk breast cancer produced long lead times before relapse (42), while a tissue-free epigenomic assay showed high specificity and prognostic value in early-stage triple-negative disease (43). Collectively, these data establish clinical validity for recurrence risk, but only selected settings have demonstrated that acting on the result improves outcomes.

## 6 Laboratory implementation and result interpretation

The preanalytical phase is a major source of error. Blood should be collected in validated tubes and processed using assay- and tube-specific workflows within the established stability window; no single centrifugation protocol is universally applicable. Plasma volume, storage conditions, freeze–thaw cycles, cfDNA input, hemolysis, and leukocyte contamination should be recorded. Matched leukocyte DNA is important for identifying clonal hematopoiesis-related variants, particularly in commonly affected genes, and bioinformatic filtering alone may not eliminate every false-positive call. Postoperative sampling should avoid the immediate period of tissue injury, when abundant non-tumor cfDNA can dilute ctDNA and reduce analytical sensitivity.

Assay selection should match the clinical and biological question. Tumor-informed methods are appropriate when adequate tumor tissue and turnaround time are available; tumor-naïve approaches may be preferable when tissue is limited or rapid deployment is required. Reports should specify the genomic territory or number of variants tracked, methylation or fragmentomic components, plasma volume, limit of detection, sample-level calling rule, leukocyte filtering strategy, and whether the result is a landmark or longitudinal interpretation. Cross-platform results should not be treated as directly interchangeable because analytical sensitivity and positivity definitions differ (10–15, 29–31, 41, 43). Table 1 summarizes the main trade-offs; neither architecture is universally superior, and results should not be treated as interchangeable across assays.

Immune testing requires equivalent rigor. PD-L1 reports should identify antibody clone, scoring system, specimen type, and cutoff. MSI/dMMR and TMB require platform-specific validation. Flow-cytometric measurement of MDSCs or lymphocyte subsets needs standardized gating, specimen handling, and reference populations. Cytokine assays require attention to collection timing, matrix effects, and multiplicity. TCR sequencing reports should distinguish diversity, clonality, expansion, and sequence-based models. A practical integrated report should present ctDNA status and kinetics first, followed by immune recognition markers, immune-suppression markers, and explicit limitations. It should avoid deterministic labels such as “responder” when the evidence supports only risk or biological stratification. TMB reports should additionally specify the assay or panel, genomic territory, variant-filtering approach, and threshold used.

## 7 Discussion

The principal value of integrating ctDNA with immune biomarkers is not the construction of a larger biomarker panel; it is the separation of two otherwise conflated questions. Is viable residual tumor detectable? And what is the state of immune recognition and escape? ctDNA addresses the first question more directly, while immune biomarkers address the second. This distinction is clinically relevant because a static tissue biomarker may not represent the current residual clone, and a circulating inflammatory signal may not be tumor specific. The proposed four-state framework is therefore conceptual and requires prospective testing to determine whether immune profiling provides incremental and clinically useful information beyond ctDNA alone.

Several controversies remain. Prognostic validity denotes association with outcome, predictive validity denotes differential treatment benefit, and clinical utility requires evidence that biomarker-guided care improves patient outcomes. First, high prognostic accuracy does not establish that biomarker-guided intervention improves survival. The negative or inconclusive experience of some MRD-triggered studies highlights the need to verify that an effective intervention exists, that the biomarker lead time is sufficient, and that the assay captures the relevant residual clone. Second, composite immune biomarkers can improve discrimination but may be difficult to reproduce across laboratories. Third, biological meaning is time dependent: early ctDNA release can reflect cell killing, while persistent or rising ctDNA more strongly indicates viable disease; similarly, immune activation can precede response, toxicity, exhaustion, or adaptive resistance.

For immunotherapy and cell-therapy research, an integrated laboratory framework offers a rational set of translational endpoints. ctDNA kinetics can quantify molecular burden, while antigen-presentation integrity, TCR expansion, circulating effector-cell persistence, cytokines, and suppressive myeloid populations can explain why burden clears or persists. The immediate priority is not universal treatment assignment, but prospective studies with synchronized plasma and immune sampling, prespecified assay thresholds, and clinically meaningful endpoints. Cross-institutional quality assessment and common reporting standards will be essential before integrated MRD–immune stratification can be used routinely.

## 8 Conclusion

ctDNA and immune biomarkers provide complementary views of molecular residual disease. ctDNA detects tumor-derived material within assay and biological limits and describes molecular kinetics; immune biomarkers describe recognition, effector competence, and escape. Their joint interpretation may distinguish biologically different MRD states that appear similar when either dimension is assessed alone, but the proposed categories remain conceptual rather than clinically validated. A result of “ctDNA not detected” indicates no detectable signal within the analytical and biological limits of the assay and does not establish absence of residual disease. Recent studies across colorectal, lung, urothelial, melanoma, and breast cancers support the clinical validity of ctDNA surveillance, while immune biomarker research supplies mechanistic context for immunotherapy-oriented stratification. Translation into routine practice now depends on analytical standardization, serial testing, transparent reporting of uncertainty, and prospective demonstration that integrated biomarker use improves patient outcomes.
